# The Spread of HIV in Pakistan: Bridging of the Epidemic between Populations

**DOI:** 10.1371/journal.pone.0022449

**Published:** 2011-07-25

**Authors:** Muhammad R. Khanani, Mehreen Somani, Sadiq S. Rehmani, Nazle M. C. Veras, Marco Salemi, Syed H. Ali

**Affiliations:** 1 Department of Microbiology, Dow University of Health Sciences, Karachi, Pakistan; 2 Department of Biological and Biomedical Sciences, Aga Khan University, Karachi, Pakistan; 3 Department of Pathology, Immunology and Laboratory Medicine, University of Florida College of Medicine and Emerging Pathogens Institute, Jacksonville, Florida, United States of America; UCL Institute of Child Health, University College London, United Kingdom

## Abstract

In the last two decades, ‘concentrated epidemics’ of human immunodeficiency virus (HIV) have established in several high risk groups in Pakistan, including Injecting Drug Users (IDUs) and among men who have sex with men (MSM). To explore the transmission patterns of HIV infection in these major high-risk groups of Pakistan, 76 HIV samples were analyzed from MSM, their female spouses and children, along with 26 samples from a previously studied cohort of IDUs. Phylogenetic analysis of HIV *gag* gene sequences obtained from these samples indicated a substantial degree of intermixing between the IDU and MSM populations, suggesting a bridging of HIV infection from IDUs, via MSM, to the MSM spouses and children. HIV epidemic in Pakistan is now spreading to the female spouses and offspring of bisexual MSM. HIV control and awareness programs must be refocused to include IDUs, MSM, as well as bisexual MSM, and their spouses and children.

## Introduction

Since the earlier reports of HIV epidemics amongst IDU communities [1], [2], in the last two decades, ‘concentrated epidemics’ have established in several high risk groups in Pakistan, including MSM [3], [4], [5]. Communities of IDUs and MSM are known to interact through overlapping risk behaviors (needle-sharing, sexual contact, etc.), which may facilitate spillover of HIV infection from IDUs into MSM, or vice versa. Indeed, such bridging phenomena have been observed in Eastern Europe, Russian Federation and Central Asia where HIV epidemics once concentrated among IDUs are now increasingly characterized by significant transmission into MSM, and subsequently, general population [6], [7]. Since MSM in Pakistan exhibit multiple high risk behaviors that overlap with those of IDUs, we hypothesized that MSM may act as ‘bridge’ for introducing the HIV infection into the general population from the IDU community, where ‘bridging’ is defined as transmission of HIV from high-risk core groups to non-core groups [6]. ‘Core’ groups comprise of individuals with high prevalence of HIV infection, having a high potential for disease transmission. In the present case, the IDUs, exhibiting practice of needle-sharing and unsafe sex, qualified as core group. The studies conducted on overlapping risk behaviors of IDU and MSM groups in Pakistan [4], [8], [9] have suggested that the widespread interlinking of the two groups carried potential for spreading the infection into general population. We recently showed evidence of a founder effect of HIV in an IDU community in Karachi, most likely brought into the community by Pakistani deportees from the Middle East [10]. Using phylogenetic analyses, we now show that the HIV epidemics amongst Pakistani MSM may be linked to the one we previously reported in IDUs. Furthermore, we show evidence that from the MSM community, the HIV epidemic is spreading into the spouses and children of bisexual MSM, thus seeding the epidemic into non high-risk populations.

## Methods

### Ethics Statement

The study was approved by the Ethical Review Committee, Aga Khan University, Karachi, Pakistan. Written informed consent was obtained from all study participants or/and their guardian.

### Subject Profile

MSM groups were identified from various parts of Karachi, and some from rural areas of Larkana and Sangar. A written informed consent was obtained along with the data on gender, age, ethnic background, occupation, marital status and high risk behaviors from the study participants prior to the collection of blood samples. In case of a participant aged less than 18 years, consent of the parent/guardian was obtained. The study subjects belonged to varying age groups ranging from 18 to 46 years of age and had different ethnic backgrounds. Information obtained through questionnaires revealed that the HIV positive participants practiced multiple high risk behaviors, including intravenous drug use (34.04%) and contact with commercial sex workers (17.02%) (Table 1). A total of 47 MSM, 15 MSM spouses and 14 MSM children samples were used for this study.

**Table 1:** Demographic details of HIV gag PCR positive participants.

Categories	MSM (n = 47)	MSM Spouse (n = 15)	MSM Children (n = 14)
**Location**			
Karachi	29 (61.7%)	3 (20.0%)	2 (14.28%)
Larkana	5(10.6%)	0	0
Sangar	2 (4.25%)	1 (6.66%)	0
Unknown	11 (23.4%)	11 (73.3%)	12 (85.71%)
**Age Groups**			
0–10 years	0	0	14(100.0%)
11–20 years	3 (6.38%)	0	0
21–30 years	20 (42.5%)	12 (80.0%)	0
31–40 years	15 (31.9%)	1 (6.66%)	0
41–50 years	6 (12.7%)	2 (13.3%)	0
Unknown	3 (6.38%)	0	0
**Marital Status**			
Married	21 (46.8%)	14 (93.3%)	NA
Single	18 (38.3%)	0	NA
Divorced	1 (2.12%)	1 (6.66%)	NA
Missing data	7 (14.9%)	0	NA
**Ethnicity**			
Balochi	1 (2.12%)	0	0
Bengal	2 (4.25%)	0	0
Barmi	0	1 (6.66%)	0
Muhajir	1 (2.12%)	0	0
Pathan	5 (10.6%)	0	0
Punjabi	9 (19.1%)	2 (13.3%)	3 (21.42%)
Sindhi	9 (19.1%)	3 (20.0%)	1 (7.14%)
Saraiki	2 (4.25%)	0	0
Urdu Speaking	1 (2.12%)	0	0
Missing data	17 (36.1%)	9 (60.0%)	10(71.4%)
**High Risk Behaviors**			
Total Drug Users	16 (34.04%)	NA	NA
Total Promiscuity	1 (2.12%)	NA	NA
Total Contact with Sex workers	8 (17.02%)	NA	NA

The frequencies of all values are given in parentheses.

### Extraction of DNA and polymerase chain reaction (PCR)

Blood samples were collected and total DNA was extracted from the buffy coat using QIAmp DNA Blood Mini Kit (250) from *Qiagen*. PCR amplification of complete *gag* of HIV-1 was performed with two sets of primers in a two-step nested PCR strategy. The primers used in the first round of PCR were GOPF (5′- CTCTCGACGCAGGACTCGGCTTGC- 3′, nt 683–706, HXB2) and GOPR (5′- CCAATTCCCCCTATCATTTTTGG-3′, nt 2382–2404). For the second round of amplification, primers GIPF (5′- GAGGCTAGAAGGAGAGAGATGGG-3′, nt 772–794, HXB2) and GIPR (5′-TTATTGTGACGAGGGGTCGTTGCC-3′, nt 2269–2292) were used [11].

The reaction mixture of 25 µl for both first and second round PCR contained 1× PCR buffer (5× Green GoTaq® Flexi Buffer, pH 8.5), 2 mM MgCl2, 400 µM dNTPs and 0.3 U of *Taq* Polymerase. The first round of PCR was performed with 0.48 pmol of primers GOPF and GOPR. Thermocycle was: denaturation at 95°C for 5 min, followed by 40 cycles of denaturation at 95°C for 1 min, annealing at 58°C for 1 min and extension at 72°C for 1 min, with a final extension of at 72°C for 15 min.

1 µl of the first-round PCR product along with 0.48 pmol of the primers GIPF and GIPR was used for the second-round PCR. Thermocycle was: denaturation at 95°C for 5 min, followed by 40 cycles of denaturation at 95°C for 1 min, annealing at 60°C for 1 min and extension at 72°C for 1 min, with a final extension of at 72°C for 15 min. The amplified products were electrophoresed on 1% Agarose gel, stained by Ethidium bromide and visualized under ultraviolet light.

### Sequencing and Phylogenetic analysis

Nested PCR products of gag gene were partially sequenced from Macrogen, Inc, Korea, using the primer GSP1 (59- CCATCAATGAGGAAGCTGC-39, nt 1400–1418, HXB2). For subtyping and further analysis, the nucleotide sequence spanning the p24 and p7 region of gag gene, nt 1577–2040, HXB2 (comprising 460–470 bp), was aligned with sequences from the Los Alamos HIV sequence database.

Maximum likelihood (ML) phylogenetic trees were inferred with PhyML program [12] [http://www.atgc-montpellier.fr/phyml/], using the GTR+G+I nucleotide substitution model, which was selected with the hierarchical likelihood ratio test described by Swofford and Sullivan [13]. Subtypes F [GenBank: AF005494 and AF075703], J [GenBank: AF082394 and AF082395], and K [GenBank: AJ249235 and AJ237807] sequences were used as outgroups. ML trees reliability was evaluated by SH-like approximate likelihood ratio test (aLRT) [14], which compares the likelihoods of the best and the second best alternative arrangements around the branch of interest. According to type I error rate (test significant | branch is not corrected) analysis, the aLRT of an interior branch is almost exact for a cut-off value higher or equal to 0.9, and can be considered robust for 0.75<p<0.9.

Neighbor-joining (NJ) genealogies were also inferred using the same substitution model with PAUP*4.0b10 program [13]. The reliability of NJ trees was evaluated by analyzing 1000 bootstrap replicates. Final trees were visualized and annotated with FigTree v.1.2.2 [http://tree.bio.ed.ac.uk/software/figtree/].

## Results

**Figure 1:**
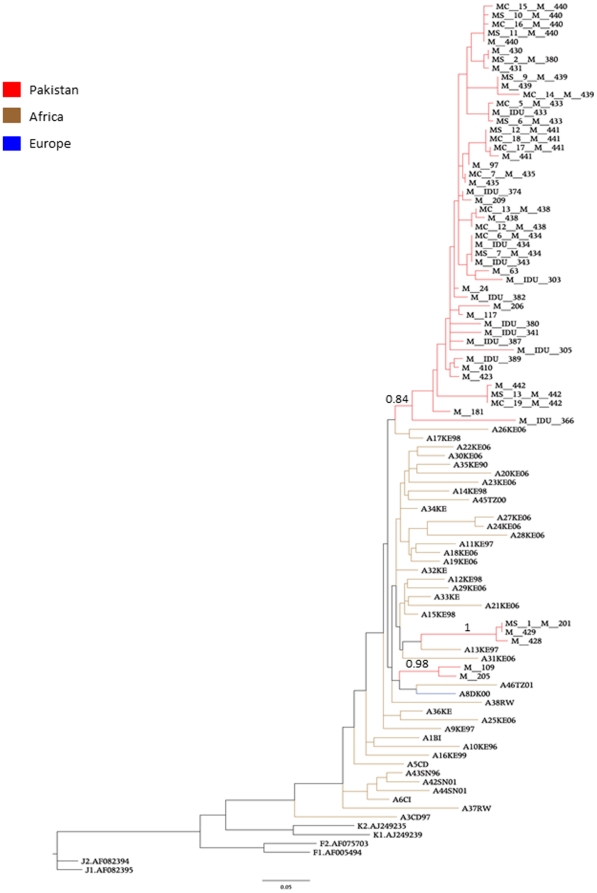
ML Phylogenetic tree of HIV-1 A Pakistani strains. ML trees where obtained with the ML method using the best fitting nucleotide substitution model. Branch lengths are scaled in nucleotide substitutions per site according to the bar at the bottom of each tree. Each tree includes the newly sequenced strains from Pakistan as well as reference strains from the HIV databases. Branches in each tree are colored according to the color legend in the figure. Pakistani sequences were labeled according to risk behavior as follows: M = male having sex with male (MSM), MS = spouses with infected partner, MC = MSM children with infected parent(s) and M-IDU = MSM who were injecting drug users. Reference strains were labeled using the HIV databases ID, which include a two letter code indicating the country of origin (http://www.hiv.lanl.gov/).

Alignment of these samples with reference sequences obtained from Los Alamos HIV database revealed that 53 (69.73%) samples were HIV-1 subtype A, 13 (17.10%) samples clustered with HIV circulating recombinant form (CRF)_AE, 2 (2.63%) samples grouped with HIV-1 subtype G and 8 (10.52%) assembled with HIV-1 CRF_AG (data not shown). Subtype specific alignments revealed several interesting patterns. The majority (90%) of subtype A Pakistani samples clustered within a monophyletic clade both in the ML (p = 0.84) (Fig. 1) and in the NJ tree (data not shown). The clade appeared to be closely related to Kenyan sequences, suggesting a limited founder effect from East Africa followed by epidemic spread in Pakistan. The remaining CRFs and subtypes showed associations from sequences from Thailand, Spain, and Senegal (data not shown).

**Figure 2:**
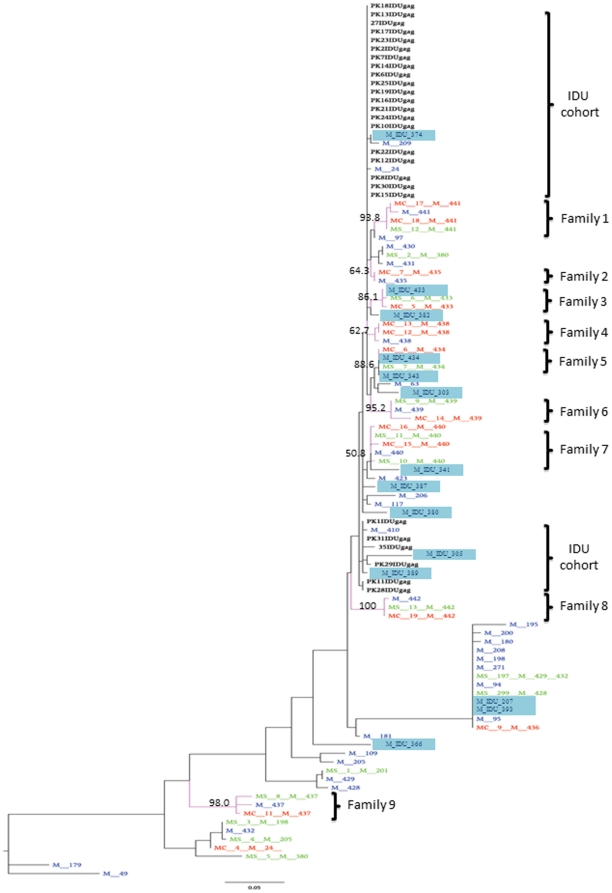
Phylogenetic analysis of HIV transmission between IDUs, MSM, and MSM families. The studied population sequences were compared to the IDU sequences obtained from a previous study, which are highlighted in black. The 47 strains from MSM and M-IDU (highlighted) are shown in blue, 15 MSM spouses (MS-) are shown in green and 14 MSM children (MC-) are shown in red. Members of each family are assigned by similar digit label following the letter prefix. The numbers along the monophyletic branches correspond to bootstrap values. Branch lengths in nucleotide substitutions per site were scaled according to the bar at the bottom of the tree.

Phylogenetic relationship was sought between the MSM *gag* sequences and those from a previously reported community of IDUs [10] (Fig. 2). Through interviews, practice of intravenous drug use was also established for certain MSM study subjects (labeled M-IDU in Fig. 2). As both the MSM and IDU communities resided in the same city and visited the same needle-exchange and healthcare centers, a possibility of HIV transmission through needle-sharing between these groups was speculated. This notion was confirmed through phylogenetic analyses of sequences from IDUs, MSM, and M-IDU. Several of the M-IDUs were part of the MSM clusters, whereas one was clustered with the 20 IDU samples, part of the cohort we have previously reported to be infected through a founder effect [10] (Fig. 2). Within this cluster, two other MSM were also found in tight association with the IDU samples. Altogether, thirteen HIV positive MSM families were analyzed for phylogeny. Out of these 13, nine families (Family 1–9, Fig. 2) showed strong association; forming nine discreet and tight phylogenetic clusters, indicating the transmission of the same virus within each family. Among the nine families studied, for two (Family 1 and Family 2) data for only father and children were available, whereas seven (Family 3 through 9) represented complete family units comprising mother, MSM-father, and the offspring. Similar phylogenetic associations were observed when the trees were reconstructed using the Neighbor Joining method or a different software, PhyML (data not shown).

## Discussion

Results we present here show that HIV epidemic in the Pakistani MSM community is represented by HIV subtypes A and G, and by CRFs AE and AG. The analysis of sequences from IDUs, MSM and MSM families revealed a possible bridging of HIV infection from the core high risk group of IDUs, through MSM and M-IDU (MSM practicing intravenous drug use), into the families of MSM. Our phylogenetic analysis implicates M-IDU to be involved in bridging the HIV transmission between IDUs and MSM, most likely through needle-sharing. The M-IDU may have contracted the virus from the IDU ‘core’ group through needle-sharing and sexually transmitted the infection to other MSM. Although it was not revealed through the interviews conducted, the possibility of sexual transmission between IDUs and MSM also exists. In the phylogenetic analysis we performed, several discreet clusters of MSM sequences containing M-IDU sequences were found, implicating M-IDU in transmitting the infection from IDU to MSM community. Close clustering of the HIV sequences in nine MSM families was observed, indicating infection of the MSM husband, wife and the offspring with the same virus. The same analysis also suggests the direction of HIV transmission to be: IDU→MSM→female spouses→children (Fig. 2). Additional analyses, incorporating time-frame of infection, would need to be performed to establish the direction of transmission more clearly.

Similar to the transmission patterns observed in Russia [6], China [15], Thailand [16], Vietnam [17], and Bangladesh [17], the epidemic of HIV in Pakistan now appears to be spreading from IDUs to MSM. As per estimates, around 0.13–0.16% of the population of Pakistan is involved in injecting drug use. Among these 125–150,000 IDUs, the prevalence of HIV on average varies from 15.4–18.3% [18]. These figures admonish that the rapidly spreading HIV epidemic in the IDU community has reached to proportions where it can now readily spill over into other high-risk groups and/or the general population. Previous reports have documented a cross-sectional picture of high-risk behaviors and HIV infection in the MSM community in Pakistan. We have found 11.36% sero-positivity for HIV among MSM [5], which is considerably higher than what has been documented earlier [4], [6], [18], [19]. This high prevalence of HIV in the MSM community indicates a rising epidemic now in a high-risk group other than IDUs. Apart from practicing unsafe sex, MSM often indulge in injection drug use [19] and can therefore serve as a likely bridge between the IDU and MSM populations, as is indicated by the current study. Additionally, in order to avoid social stigma, MSM in Pakistan are known to exhibit bisexuality; often legally married to a female spouse while maintaining sexual contact with fellow MSM [19]. As a consequence, MSM population can serve to bridge the transmission of HIV into non-high risk general population, comprising the MSM female spouses and their children.

In light of the findings we report here, IDUs, M-IDUs, homo- and bi-sexual MSM, their female sexual partners, and offspring, need to be recognized as special focus groups for programs implementing screening, treatment and awareness of HIV. As antiretroviral therapy (ART) becomes accessible and affordable in Pakistan, the children and spouses we recruited for our study are now under treatment with ART. With improvement in the effectiveness of ART in the past years, life span of HIV patients has considerably increased [17]. Chances are, therefore, that the children of MSM subjects we studied will live to see adulthood and will likely produce offspring, transmitting infection to the next generation. In this scenario, a need to implement programs for HIV screening and for raising awareness is imperative for this infected segment of the population. It is important that the spouses and children of MSM be treated as a special focus group requiring awareness about how to control transmission of HIV into their sex partners and the next generation of children.
